# A Pilot Study Exploring the Association of Entacapone, Gut Microbiota, and the Subsequent Side Effects in Patients With Parkinson’s Disease

**DOI:** 10.3389/fcimb.2022.837019

**Published:** 2022-04-05

**Authors:** Shih-Chen Fu, Chung-Han Lee, Yi-Chen Hsieh, Pei-Hua Wu, Sheng-Hsuan Lin, Hsiuying Wang

**Affiliations:** ^1^ Institute of Statistics, National Yang Ming Chiao Tung University, Hsinchu, Taiwan; ^2^ Institute of Data Science and Engineering, National Yang Ming Chiao Tung University, Hsinchu, Taiwan

**Keywords:** Parkinson’s disease, microbiome, entacapone, constipation, drowsiness, levodopa

## Abstract

**Background and Aims:**

Entacapone, one of the most common drugs distributed among patients with Parkinson’s disease, is a peripherally acting catechol-O-methyltransferase (COMT) inhibitor that is used in addition to levodopa to control symptoms. However, there have been negative effects reported against entacapone, namely, gastrointestinal (GI) problems and drowsiness. In this pilot study, we aim to examine the hypothesis that the discomfort induced by entacapone might be originated from the shift of microbial composition by adjusting the effect of levodopa.

**Methods:**

The population in this pilot study consisted of 13 PD patients treated with levodopa only and 11 with both levodopa and entacapone. The 16S rRNA gene sequence data were processed, aligned, and categorized using the DADA2. Alpha diversity indices for Observed, Chao1, Shannon, and Simpson metrics were calculated with Phyloseq 1.32.0. Dissimilarities were calculated using unweighted unique fraction metrics (Unifrac), weighted Unifrac, and Canberra distance. Functional differences were calculated by PICRUSt2 based on the KEGG database.

**Results:**

Results of 16S rRNA sequencing analysis showed that while entacapone did not influence the species richness, the composition of the microbial community shifted considerably. Relative abundances of bacteria related to constipation and other GI disorders also altered significantly. Functional enrichment analysis revealed changes in the metabolic activity of alanine, aspartate, and glutamate. These amino acids are related to common side effects of entacapone such as auditory hallucinations, fatigue, and nightmare.

**Conclusion:**

Our findings provide testable hypothesis on the cause of unpleasant side effects of entacapone, which in the long run could possibly be reduced through gut microbiota manipulation.

## Introduction

Parkinson’s disease (PD) is one of the most common neurodegenerative disorders in the world which currently cannot be prevented or cured (Lebouvier et al., 2009; Wang, 2021). Its clinical features are akinesia, bradykinesia, rigidity, and tremors, which are known to be directly related to the progressive death of dopaminergic neurons (Xia and Mao, 2012). Levodopa (LD), a substance that could cross the blood–brain barrier and further increase brain dopamine concentrations, is currently the most effective symptomatic agent for the treatment of PD (Haddad et al., 2018). The use of LD is often coupled with entacapone, a catechol-O-methyltransferase (COMT) inhibitor, to increase its effectiveness and to reduce the risk of motor complications in PD patients (Schrag, 2005).

Adjunctive use of entacapone with LD in PD is beneficial by preventing catechol-O-methyltransferase from breaking down and metabolizing LD, which extends and stabilizes the clinical response to LD (Kaakkola, 2000). Although the addition of entacapone combined with LD is beneficial, several bothersome side effects come along with the treatment such as abdominal pain, constipation, diarrhea, nausea, etc. Some may even experience serious side effects that require immediate medical attention (Chong and Mersfelder, 2000). In a meta-analysis done with 14 studies to compare the effectiveness and safety of adjuvant treatment of entacapone in PD, a trend towards increased patient withdrawal was found due to intolerable adverse events. Patients on entacapone had a higher frequency of adverse events than those on placebo (Li et al., 2017). Although it is hypothesized that these adverse events may be related to the increase of dopamine caused by entacapone in the corpus striatum (Li et al., 2017), the underlying mechanism is still unclear.

Research have shown that human microbiome plays a critical role in the development and progression of major human diseases such as gastrointestinal disorders, metabolic diseases, psychological diseases, etc. (Wang et al., 2017). Human cohort studies have also provided insights between use of drugs and altered microbial composition and functional profiles (Zhernakova et al., 2016; Jackson et al., 2018; Vila et al., 2020). Evidence showed that there is a direct metabolizing effect of bacteria by entacapone (Weersma et al., 2020). Conversely, entacapone also inhibits the growth rate of several bacterial species (Maier et al., 2018). Despite the evidence showing complicated bidirectional interactions between entacapone and gut microbes, the studies were either done *in vitro* or *in vivo* without eliminating the effect of LD (Unger et al., 2016; Maier et al., 2018; Weis et al., 2019; Zimmermann et al., 2019).

In this study, we investigated the potential effect of entacapone alone on the alteration of microbial composition and the association of this alteration with the ensued side effects. We present a case–control study where microbiota in PD patients treated with LD only (PD_L) and patients treated with entacapone in addition to LD (PD_LE) are analyzed. We ensured the study groups did not differ with respect to possible confounders and then employed tools for bacterial identification, evaluation of ecological diversities of the microbiome, and functional predictions.

## Methods

### Participant Recruitment and Data Collection

We adapted our data from the study of Weis et al. (2019), in which 34 PD patients and 25 healthy controls were recruited. Among the cohort, 13 patients (6 women, 7 men) treated with LD only (PD_L) and 11 patients (4 women, 7 men) treated with both LD and entacapone (PD_LE) were selected for our study. Both groups were age and sex matched. At the time of sampling, the mean age of PD_L group was 70.6 (sd = 8.8) years and 69.9 (sd = 5.39) years for the PD_LE group. Mean duration of the disease was 77.9 (sd = 50.9) and 120 (sd = 54.3) months for PD_L and PD_LE respectively. Sequencing library preparation of the V4 and V5 region of the bacterial 16S rRNA genes were performed using the 16S-specific primers 520 F (5′-AYTGGGYDTAAAGNG-3′) and 926 R (5′-CCGTCAATTCMTTTRAGTTT-3′). Unique, custom made index barcodes with sequencing adapter (CCATCTCATCCCTGCGTGTCTCCGACTCAG) were added to the amplicon targets. Details of diagnostic criteria for PD, fecal sample collection process, DNA isolation, library preparation and sequencing can be found in the previous reference (Weis et al., 2019).

### Data Availability and Ethical Statement

Sequences analyzed in this study are accessible at the European Nucleotide Archive (ENA) under the accession number PRJEB30615. Subject data can be found in the supplementary information files in the paper of Weis et al. All data are open-access and de-identified. No ethical approval is required.

### Processing of 16s rRNA Sequence Data

The 16S rRNA gene is highly conserved in bacteria. As a result, it is highly suited as a target gene for DNA sequencing for bacterial identification. The 16S rRNA gene sequence data was downloaded at the European Nucleotide Archive (ENA) under the accession number PRJEB30615 (Weis et al., 2019). Each sample was sequenced four times (paired-end reads) and we merged the sequences of four independent runs into a single dataset for all samples. The combined sequence reads were then processed with Trimmomatic v0.39 (Bolger et al., 2014) to remove the adaptors. The outputs were then processed, aligned, and categorized using the DADA2 1.16 (Callahan et al., 2016) pipeline. In brief, sequence reads were first filtered using recommended parameters of DADA2. Filtered reads were then de-replicated and de-noised using DADA2 default parameters. After building the amplicon sequence variant (ASV) table and removing chimeras, taxonomy was assigned using SILVA v132 natively implemented in DADA2. We used the addSpecies function in DADA2 to add species-level annotation with SILVA as reference. Sequence counts were normalized to relative abundances (Calculated by dividing the number of sequences that were assigned to a unique ASV by the total sequence count in the sample, which is also termed as Total-Sum Scaling) for later use. In addition to scaling, rarefying is another popular approach for normalization in literatures. However, rarefying was not adapted in this study due to the limitations of potentially reducing statistical power and the restriction of sample size of our dataset.

### Statistical Analyses of 16s rRNA Sequence Data

Alpha diversity indices for Observed features, Chao1, Shannon, and Simpson metrics (Chao, 1984; Magurran, 1988; Rosenzweig, 1995) were calculated with Phyloseq 1.32.0 (McMurdie and Holmes, 2013). Dissimilarities (distance) between the microbiomes of PD_LE and PD_L samples were calculated using the following metrics to ensure that the choice of metrics did not affect the results: unweighted unique fraction metrics (Unifrac), weighted Unifrac (Lozupone et al., 2011), and Canberra distance (Lance and Williams, 1967). Beta diversity indices for weighted and unweighted unifrac were calculated with Phyloseq 1.32.0 (McMurdie and Holmes, 2013). Canberra distance was calculated with vegan 2.5.7. The differences between PD_LE and PD_L were tested. P-values for alpha diversity were calculated with ANOVA using stats 4.0.5 and beta diversity with ADONIS using vegan 2.5.7. The identification of significantly different phylum and genus between PD_LE and PD_L was performed using t-test. Variable comparison between the two groups were conducted by Fisher’s exact test and Wilcoxon rank sum test.

### Functional Enrichment Analysis of Predicted Metagenomes

We used Phylogenetic Investigation of Communities by Reconstruction of Unobserved States (PICRUSt2) version 2.4.1 (Douglas et al., 2020) to infer metagenome composition in the samples, following the recommended pipeline of normalizing ASVs by copy number (to account for differences in number of copies of 16S rRNA between taxa), predicting functions using Kyoto Encyclopedia of Genes and Genomes (KEGG) (Kanehisa and Goto, 2000) orthologs, and grouping predicted pathways by KEGG hierarchical level 3. We tested PD_L and PD_LE differences for all metabolic pathways presented in all of our samples (N = 116 pathways) using the Statistical Analysis of Metagenomic Profiles (STAMP) software version 2.1.3 (Parks and Beiko, 2013). We compared PD_L versus PD_LE using White’s non-parametric t-test (two sided, replication = 1,000), with a Storey FDR <.05 as a cutoff for significance.

## Results

### Processing 16S Reads Using DADA2

Patients treated with LD and entacapone (n = 11) and those treated with LD only (n = 13) from Weis et al. (2019) were selected for our study. The general characteristics of these participants are summarized in **Table 1**. We downloaded the V4 and V5 regions of the bacterial 16S rRNA sequencing data (which was sequenced using an Ion Torrent PGM platform.) of the 24 samples to further characterize the microbiome. A total of 15,019,733 sequences with a mean of 625,822 sequences per sample (min: 85,858; max: 2,710,753 sequences) was obtained. The average read length was 293.28 base pairs, totaling 4.41 G bases. After filtering, denoising, and removing chimeras, we retained 11,608,066 (77.29% of initial) reads. We then assigned taxa to these reads. A total of 11,432 ASVs were identified, in which 15 phyla and 255 genera were assigned. The number and all detailed information of all taxonomic levels were in the **Supplementary Materials**.

**Table 1:** Comparing patients receiving entacapone and those without on available variables.

	P_LE (N = 11)	P_L (N = 13)	P-value
**Sex**			
Female	4 (36.4%)	6 (46.2%)	0.697
Male	7 (63.6%)	7 (53.8%)	
**Age**			
Mean (SD)	69.9 (5.39)	70.6 (8.80)	0.431
Median [Min, Max]	69.0 [60.0, 77.0]	74.0 [50.0, 81.0]	
**Calprotectin**			
Negative	4 (36.4%)	8 (61.5%)	0.414
Positive	7 (63.6%)	5 (38.5%)	
**Smoker**			
No	11 (100%)	12 (92.3%)	1
Yes	0 (0%)	1 (7.7%)	
**Constipation**			
No	7 (63.6%)	11 (84.6%)	0.357
Yes	4 (36.4%)	2 (15.4%)	
**Disease duration months**			
Mean (SD)	120 (54.3)	77.9 (50.9)	0.0611
Median [Min, Max]	108 [57.0, 228]	72.0 [14.0, 156]	
**Hoehn Yahr stage**			
2	3 (27.3%)	4 (30.8%)	1
3	6 (54.5%)	6 (46.2%)	
4	2 (18.2%)	3 (23.1%)	
**Phenotype**			
E	4 (36.4%)	7 (53.8%)	0.0662
HR	7 (63.6%)	3 (23.1%)	
T	0 (0%)	3 (23.1%)	
**Appendectomy**			
No	9 (81.8%)	6 (46.2%)	0.105
Yes	2 (18.2%)	7 (53.8%)	
**Family history for neurodegenerative disorders**			
No	8 (72.7%)	9 (69.2%)	1
Yes	3 (27.3%)	4 (30.8%)	
**L-dopa dose**			
1	3 (27.3%)	6 (46.2%)	0.697
2	7 (63.6%)	6 (46.2%)	
3	1 (9.1%)	1 (7.7%)	
**Other GI symptoms**			
No	8 (72.7%)	11 (84.6%)	0.63
Yes	3 (27.3%)	2 (15.4%)	

### Metadata Comparison Between Patients Treated With and Without Entacapone

Microbiome can be potentially affected by numerous factors. If the distribution of such variable differs between PD_LE and PD_L, then it is possible to find a significant difference in microbiota between the study groups that is simply an artifact of the related variable. Given the reason above, we have tested the data on all variables collected. If the difference between P_LE and P_L was significant, the variable would be considered as a potential confounder. Our results showed that none of the 12 variables (sex, age, calprotectin, smoker, constipation, disease duration months, Hoehn Yahr stage, phenotype, appendectomy, family history for neurodegenerative disorders, LD dose, other GI symptoms) were significantly different between P_LE and P_L (**Table 1**), indicating that these variables did not confound the causal relationship between entacapone and the following microbiome change.

### Structural Diversity Measures

**Figure 1:**
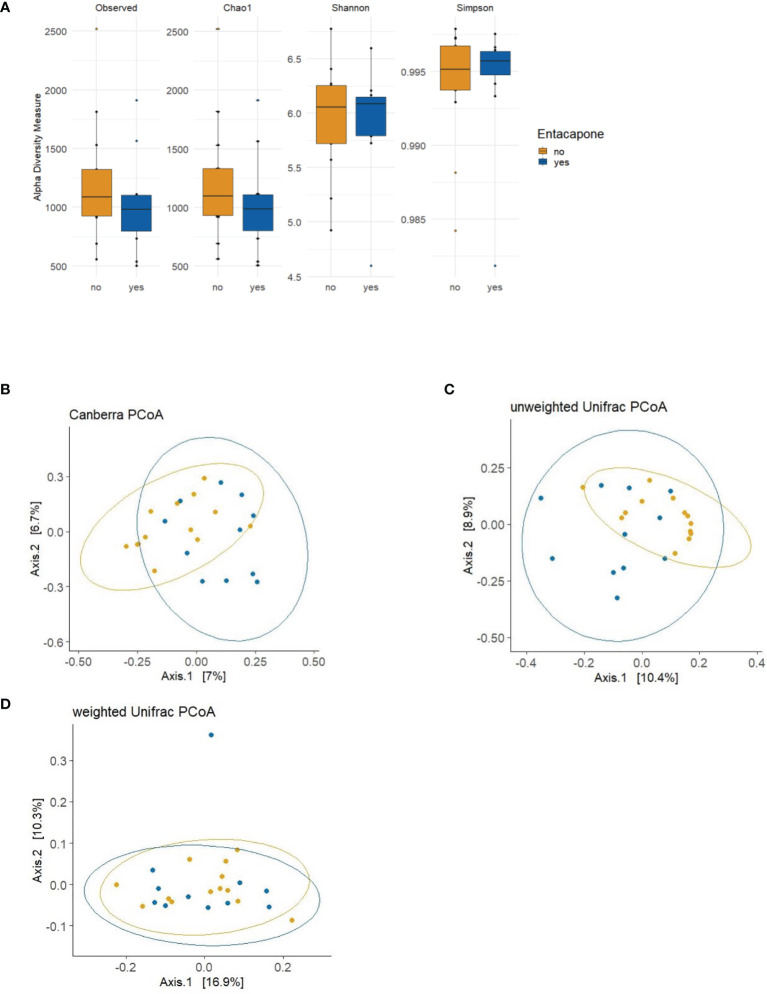
Alpha and beta diversity plots to visualize the difference in microbiota structure between the study groups PD_LE and PD_L. **(A)** The boxplots show the alpha diversity of the bacterial communities by means of observed amplicon sequence variants (ASVs), and Chao1, Shannon, and Simpson indexes. Median, and also lower and upper quartiles are shown on the plots. The PCoA plots show the following 3 distance measures: Canberra **(B)**, unweighted unique fraction metric (UniFrac) **(C)**, and weighted UniFrac **(D)**. Statistically significant differences were found between the two groups using Canberra (ADONIS; p = 0.0025; R^2^ = 0.0546; perm = 9999) and unweighted UniFrac distance metrics (ADONIS; p = 0.004; R^2^ = 0.0628; perm = 9999). Blue: PD_LE, orange: PD_L. Each dot represents an individual sample. Ellipses are drawn at 95% confidence intervals.

We compared the overall taxonomic diversity between PD_L and PD_LE using different metrics of alpha diversity that incorporate species richness and evenness. We estimated observed features (i.e., number of ASVs), and the Chao1, Shannon and Simpson indices from the ASV table. None of the measures mentioned above were significantly different between the groups (*p*_Observed_ = 0.3348; *p*_Chao1_ = 0.3384; *p*_Shannon_ = 0.9736; *p*_Simpson_ = 0.8675) (**Figure 1A**). Non-parametric method (Kruskal–Wallis test) generated similar results, which was provided in the **Supplementary Material**. We next applied both phylogenetic (UniFrac) and non-phylogenetic (Canberra) methods to calculate the dissimilarities (distance) between the microbiomes of PD_LE and PD_L samples. In contrast to alpha-diversity, we found a significant change in community structure using Canberra distance metric (*p*_Canberra_ = 0.0025). Using principle coordinate analysis (PCoA) based on Canberra distances, we found that the two highest-ranked dimensions, PCo1 and PCo2, explained 7 and 6.7% of variance respectively (**Figure 1B**). Significant difference was also found between the groups in the unweighted Unifrac measure (*p*_Unweighted-unifrac_ = 0.004). The two highest-ranked dimensions, PCo1 and PCo2, explained 10.4 and 8.9% of variance respectively (**Figure 1C**). No significant difference was found in weighted UniFrac measure between the groups (*p*_Weighted-unifrac_ = 0.1945) (**Figure 1D**).

### Identification of Taxa That Differed Between Patients Treated With and Without Entacapone

**Figure 2:**
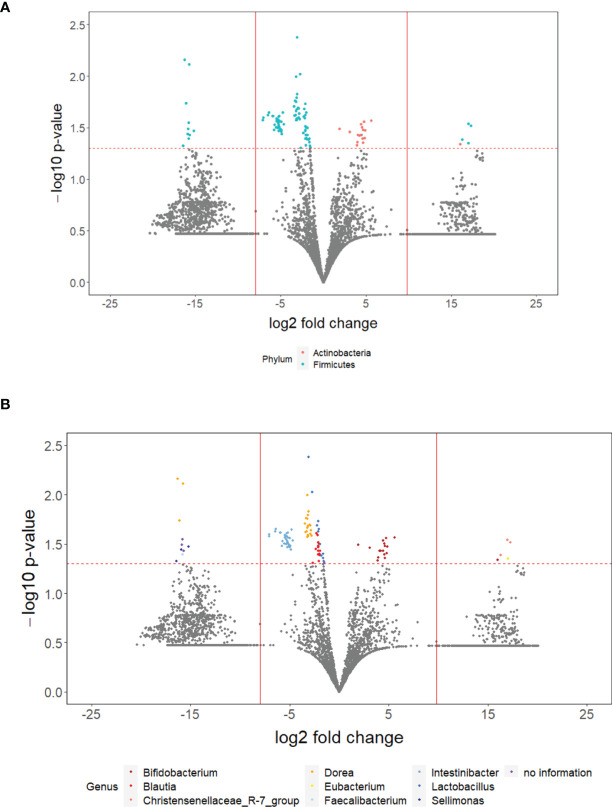
The overall difference of gut microbiota between PD_LE and PD_L individuals was assessed. A negligible number (1.635235 ∗ 10^−9^) was added to the abundance of every ASV to avoid the value of magnitude of fold change being infinitely large or small. This number was generated based on the one hundredth of the minimal relative abundance. Each point represents an ASV with its magnitude fold change in relative abundance (log2 of PD_LE/PD_L) on the x-axis and the value of statistical significance (−log10 of p-value) on the y-axis. The dashed red line shows where p = 0.05 with points above the line having p <0.05 and points below the line having p > 0.05. Significant ASVs are colored based on phylum **(A)** and genus **(B)**. Points outside of the solid lines are ASVs with mean abundance of 0 in either PD_LE (left) or PD_L (right) group.

**Figure 3:**
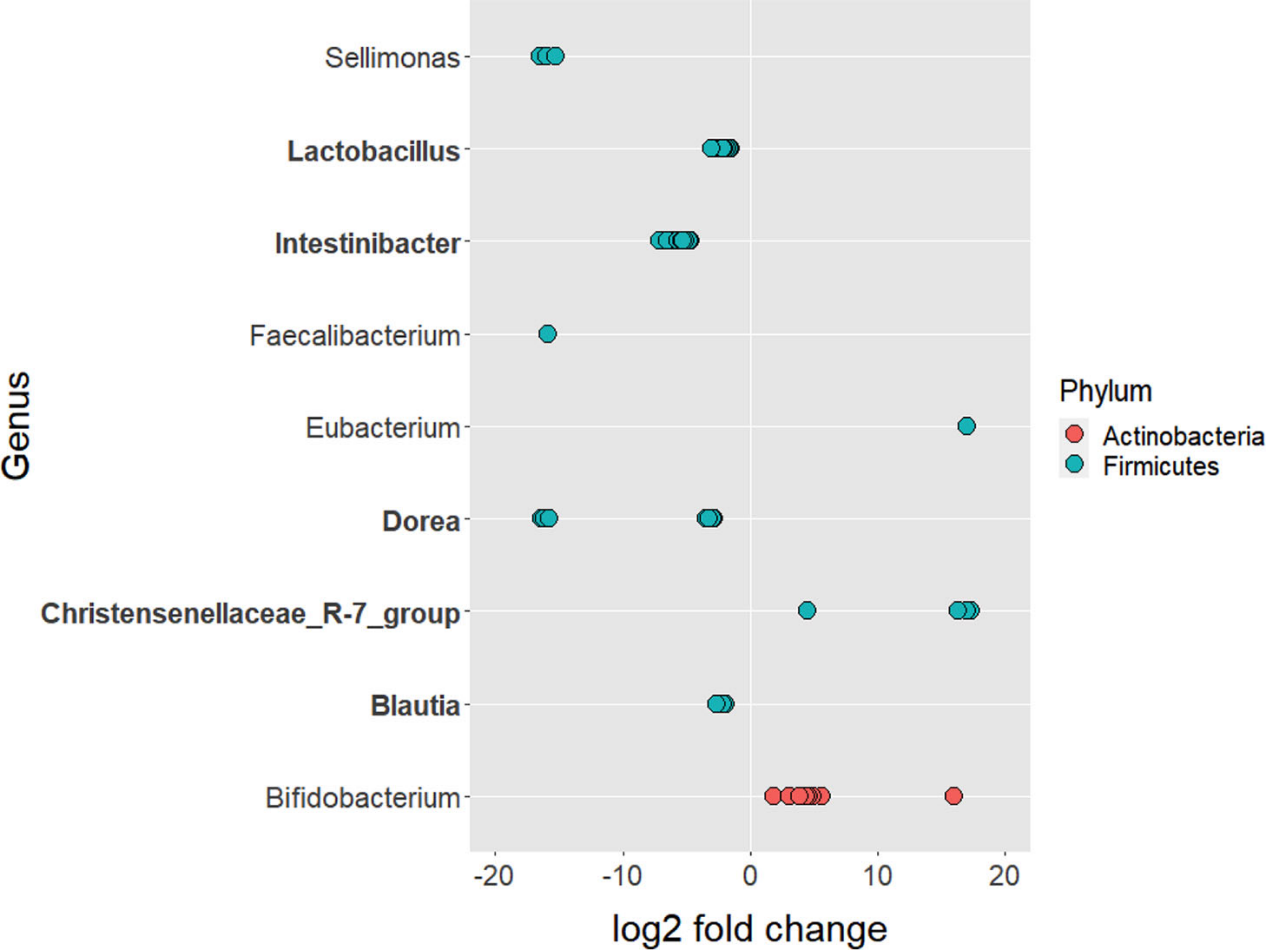
ASVs that significantly differed in abundance before and after the treatment of entacapone. 108 ASVs were identified as significantly different after the treatment of entacapone. 3 ASVs that were not assigned taxonomy to the genus level had a decrease in abundance. They were removed for plotting purposes. Genus and phylum level classification of the significant ASVs are provided in the plot. Genus in bold are unique to our study when compared with Weis et al. (2019).

Out of 11,432 ASVs detected in both PD_LE and PD_L, 108 were significantly different in relative abundance. Among the significant ASVs, 17 (15.7%) were classified as Actinobacteria and 91 (84.3%) were classified as Firmicutes. These two taxa were also the first two top phyla detected after running DADA2. Apart from the 3 ASVs without genus information, we were able to identify the rest 105 significant ASVs in 9 genera. It is worth noting that of all the significant ASVs, 86 (79.63%) were decreased and 22 (20.37%) were increased. All of the decreased ASVs were Firmicutes and 77.27% of the increased ASVs were *Actinobacterium* (**Figure 2A**). The increased genera were *Eubacterium*, *Christensenellaceae_R-7_group*, and *Bifidobacterium*. The decreased genera were *Sellimonas*, *Lactobacillus*, *Intestinibacter*, *Faecalibacterium*, *Dorea*, *Blautia* (**Figures 2B**, **3**). Only 4 significant ASVs were classified in species level (*Intestinibacter bartlettii*, *Dorea longicatena*, *Blautia obeum*). Among the bacteria that were significantly different in abundance between PD_LE and PD_L, 5 genera were unique to our study when comparing to Weis et al. (2019). These were *Lactobacillus*, *Intestinibacter*, *Dorea*, *Christensenellaceae_R_7_group*, and *Blautia* (**Figure 3**). ANCOM was also conducted for sensitivity analysis and the results were provided in the **Supplementary Material**. The aforementioned 9 genera with additional 5 genera (Agathobacter, Clostridium_sensu_stricto_1, Lactonifactor, Romboutsia, and Streptococcus) were found significantly different between the groups.

### Functional Prediction

**Figure 4:**
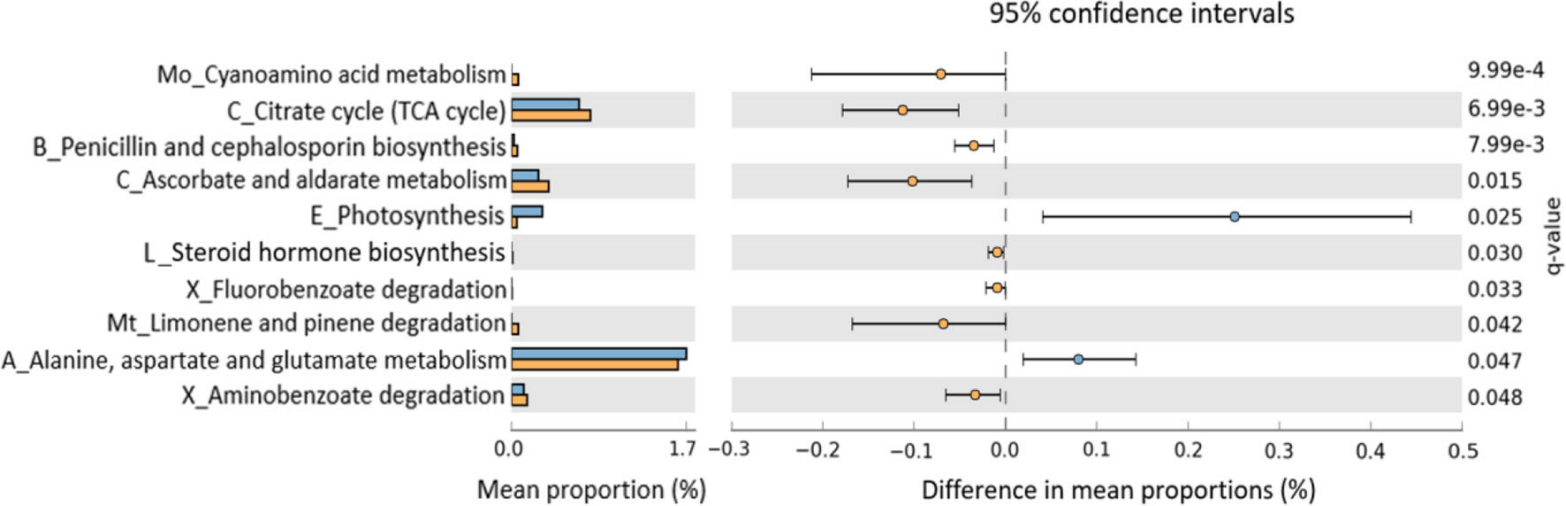
Predicted functional differences between PD_L and PD_LE microbiomes. A total of 10 metabolic pathways differed significantly between patients treated with and without entacapone. Pathways that were more abundant in PD_LE are on the positive side (blue circle with 95% confidence interval). Pathways that were more abundant in PD_L are on the negative side (orange circle). q-value, the Storey false discovery rate (FDR)-corrected *P-*value. Mean proportions are shown in stacks for PD_LE (blue) and PD_L (orange). Differences in mean proportions: mean proportion in PD_LE minus mean proportions in PD_L. Only metabolic pathways at the Kyoto Encyclopedia of Genes Genomes (KEGG) hierarchical level 1 were investigated to limit inclusion of nonbacterial pathways. Tests were conducted at KEGG hierarchical level 3, which included 116 pathways present in all samples. The letter(s) in front of each pathway name indicates the KEGG hierarchical level 2 for that pathway (A, amino acid metabolism; B, biosynthesis of other secondary metabolites; C, carbohydrate metabolism; E, energy metabolism; L, lipid metabolism; Mo, metabolism of other amino acids; Mt, metabolism of terpenoids and polyketides; X, xenobiotics biodegradation and metabolism); TCA, tricarboxylic acid.

To evaluate functional differences in the microbiomes of PD_LE versus PD_L, we used PICRUSt2 (Douglas et al., 2020), a computational tool that allows using 16S rRNA amplicon data to predict the functional potential of a bacterial community based on the KEGG database. We then tested the difference between PD_LE versus PD_L using STAMP. Among the 116 metabolic pathways tested, 10 were significantly different between PD_LE versus PD_L (**Figure 4**). According to the KEGG hierarchical level 2 classification, the 10 pathways are involved in carbohydrate metabolism, energy metabolism, lipid metabolism, amino acid metabolism, metabolism of other amino acids, metabolism of terpenoids and polyketides, biosynthesis of other secondary metabolites, xenobiotics biodegradation and metabolism.

## Discussion

Being a common medication used in combination with LD for the treatment of Parkinson’s disease, microbiologists have gained interests in the interactions between entacapone and gut microbiome, resulting in research that has been conducted in the related field (Unger et al., 2016; Weis et al., 2019; Grün et al., 2020). Yet most of these *in vivo* studies overlooked the impact of LD on the microbial composition (Weis et al., 2019; Cirstea et al., 2020), which led to findings that could possibly be associated with LD instead of entacapone. *In vitro* studies such as high-throughput drug screening have also shown significant effect of entacapone on gut microbiome (Maier et al., 2018; Zimmermann et al., 2019). However, the selected bacteria only partially recapitulate the human microbiota. These test results do not fully represent the alteration of microbiome in the human gut. Because of the limitations in previous studies, the impact on microbiome solely by entacapone treatment in PD patients is still unclear. To the best of our knowledge, this is the first study investigating the influence of entacapone *via* 16S rRNA gene sequencing by adjusting the effect of LD on the human microbiota and examining how these microbial signatures may associate with adverse reactions to entacapone.

Our results revealed that entacapone alone had no effect on phylogenetic diversity and species richness; however, it was a major effect factor for the phylogenetic composition of these samples. The majority of bacteria significantly increased and decreased in abundance were Actinobacteria and Firmicutes respectively. Several GI disorders have been proved to be associated with the imbalance of bacteria under these phyla (Passos and Moraes-Filho, 2017; Dieterich et al., 2018; Binda et al., 2018; Rinninella et al., 2019), suggesting the possibility of their mediating effects on the relationship between entacapone treatment and the subsequent side effects. Significant differences in the metabolism between PD_LE and PD_L were suggested by the overrepresentation and underrepresentation of the predicted KEGG pathways associated with different metabolic process and biosynthesis in either group.

Several findings regarding the changes in the microbiome after entacapone treatment deserve consideration. First, our findings agree with the experimental results described below, suggesting the mediating role of microbial change towards side effects, especially constipation [such as *Faecalibacterium prausnitzii*, *Bifidobacterium*, *Lactobacillus*, and *Eubacterium* (Zoppi et al., 1998; Nourrisson et al., 2014; Mirghafourvand et al., 2016; Cao et al., 2017)], caused by the intake of entacapone. Recent studies using 16S rRNA-based microbiota profiling have demonstrated dysbiosis of gut microbiota in constipation (Ohkusa et al., 2019). Although the findings are inconsistent and currently no consensus exists, studies in constipation have found significant inter-individual differences between healthy individual and patients (Zhu et al., 2014; Mancabelli et al., 2017; Li et al., 2020). Interestingly, results of alpha-diversity analysis from these studies suggested similar community richness and diversity between the two groups (Li et al., 2020), which were consistent with our findings. Evidence from a mouse study showed the causal relationship between dysbiosis and chronic constipation. Mice received fecal microbiota from patients with constipation produced constipated features such as reduction of intestinal motility, lower frequency of pellet expulsion, less fecal water content, etc. (Cao et al., 2017). A major alteration in the microbial community was also found when compared to mice receiving fecal microbiota from healthy individuals (Cao et al., 2017). Their proportion of Firmicutes and the relative abundance of Lactobacillus were both significantly lower, which could also be seen in PD_LE in our study.

A second key observation from our study was that we not only confirmed the previous reported bacteria associating with COMT-inhibitors, we have also found unique microbial signatures that are highly related to GI disorders. Results from our analysis showed that 86.16% of the significantly decreased ASVs were classified in the Firmicutes phylum in PD_LE. At genus level, the relative abundance of *Faecalibacterium* decreased by 66.08% and *Bifidobacterium* doubled when comparing PD_LE to PD_L. These results corroborate with earlier reports (Unger et al., 2016; Weis et al., 2019; Grün et al., 2020), suggesting that entacapone itself is able to redistribute bacterial community profile in PD patients. Studies done by real-time quantitative PCR showed that the abundance of *Faecalibacterium prausnitzii* significantly reduced in PD patients on entacapone when compared to controls or participants without COMT-inhibitors (Unger et al., 2016; Grün et al., 2020). In another study conducting 16S rRNA analysis, the relative abundance of *Faecalibacterium* also significantly decreased in patients treated with entacapone when compared to the controls (Weis et al., 2019). On the other hand, increasing relative abundance of *Bifidobacterium* was seen in PD patients on a COMT-inhibitor therapy (Aho et al., 2019). Interestingly, the significant decrease of *F. prausnitzii* and increase of *Bifidobacterium* were both found in the fecal samples of constipated individuals (Zoppi et al., 1998; Nourrisson et al., 2014).

On comparison of our data with the study done by Weis et al. (2019), we have revealed several genera that were specifically altered by entacapone in addition to their findings. The altered concentrations of these bacteria were associated with individuals suffered from gastrointestinal disorders, mainly constipation. For example, the deficiencies of *Lactobacillus* were involved in reduced frequency of bowel movements in pregnant woman (Mirghafourvand et al., 2016). Intestinal abundance of *Lactobacillus* is positively correlated with Crohn’s disease patients (Wang et al., 2014; Lewis et al., 2015). Another study showed the increase of *Dorea* could alleviate symptoms in constipated mice (Wang et al., 2019). Species under *Eubacterium* increased significantly in mice underwent fecal microbiota transplant from constipated participants (Cao et al., 2017).

Results from functional prediction suggested that there was an increased activity in amino acid metabolism such as alanine, aspartate, and glutamate. Accumulating evidences have indicated that the dysregulation of these amino acids might involve in the pathology of fatigue, which is one of the major side effects of entacapone (Marquezi et al., 2003; Stout et al., 2007; Li et al., 2020). Whether entacapone induced microbiome alteration leads to dizziness and drowsiness *via* regulating the metabolic pathways of the above-mentioned amino acid is worth future investigation.

Several limitation merits to be noticed. First, our results do not prove causality between entacapone, microbiota, and the side effects that may ensue due to our cross-sectional data and the small sample size (the microbial differences between patients with constipation and without constipation, with GI or without GI disorder are provided in the **Supplementary Material**). However, the correlations are plausible and worth future investigation. To further understand the causal role of gut microbiota in the development of side effects, mediation analysis of properly designed longitudinal studies with sufficient amount of sample size would be highly recommended. Second, information such as medication history or diet habit was not provided in the original dataset. In future studies, all factors with potential to affect microbiome should be measured and correctly adjusted. Third, due to the restriction of sample size, several important factors such as disease duration were not adjusted. It is worth to note that patients with entacapone had longer disease duration, despite not significantly different between P_LE and P_L groups. This phenomenon could be partially explained by the fact that patients with longer disease duration had higher prevalence of levodopa resistance and were more likely to be prescribed entacapone. Disease duration should be adjusted in future studies when dataset with larger size is available. Fourth, as far as we know, mechanisms which explained the association between certain bacteria, such as *Intestinibacter*, and constipation or other GI disorders have not been investigated by previous literature. However, it was reported that metformin, a type 2 diabetes medication and known as its severe gastrointestinal adverse effects, also significantly lowers *Intestinibacter* abundance in colon microbiota. Based on the findings, researchers proposed a hypothesis that *Intestinibacter* might subsequently play a role in inducing gastrointestinal adverse effects (Olgun, 2017; Bryrup et al., 2019). Our finding with *Intestinibacter* slightly supported this hypothesis and more sophisticated studies are required to disentangle this question. Finally, the sample size is relatively small, leading to lower statistical power and less confounders that could be adjusted. Therefore, this study should be viewed as a pilot study. All conclusions drawn from this study should be examined by studies with larger sample size and with more sophisticated design and analysis.

In conclusion, our findings provide testable hypothesis on the cause of unpleasant side effects of entacapone, which in the long run could possibly be reduced through gut microbiota manipulation.

## Data Availability Statement

Publicly available datasets were analyzed in this study. This data can be found here: European Nucleotide Archive (ENA) under the accession number PRJEB30615.

## Author Contributions

SF came up with the original idea. SF, YH, and PW set up and performed the bioinformatics procedures. CL, YH, and PW conducted data analysis. SF wrote the first version of manuscript. SF, SL, and HW contributed to the paper. All authors listed have made a substantial, direct, and intellectual contribution to the work and approved it for publication.

## Funding

This research was supported by The Ministry of Science and Technology, 107-2118-M-009 -002-MY2 (for SF and HW) and 111-2628-B-A49 -007- (for CL, YH, PW and SL), Taiwan.

## Conflict of Interest

The authors declare that this research was conducted in the absence of commercial or financial relationships that could be construed as potential conflicts of interest.

## Publisher’s Note

All claims expressed in this article are solely those of the authors and do not necessarily represent those of their affiliated organizations, or those of the publisher, the editors and the reviewers. Any product that may be evaluated in this article, or claim that may be made by its manufacturer, is not guaranteed or endorsed by the publisher.
