# Supplementary figures and images for: A Pilot Study Exploring the Association of Entacapone, Gut Microbiota, and the Subsequent Side Effects in Patients With Parkinson’s Disease
Source: Front Cell Infect Microbiol. 2022 Apr 5;12:837019. doi: 10.3389/fcimb.2022.837019 (PMC9022099; doi:10.3389/fcimb.2022.837019)

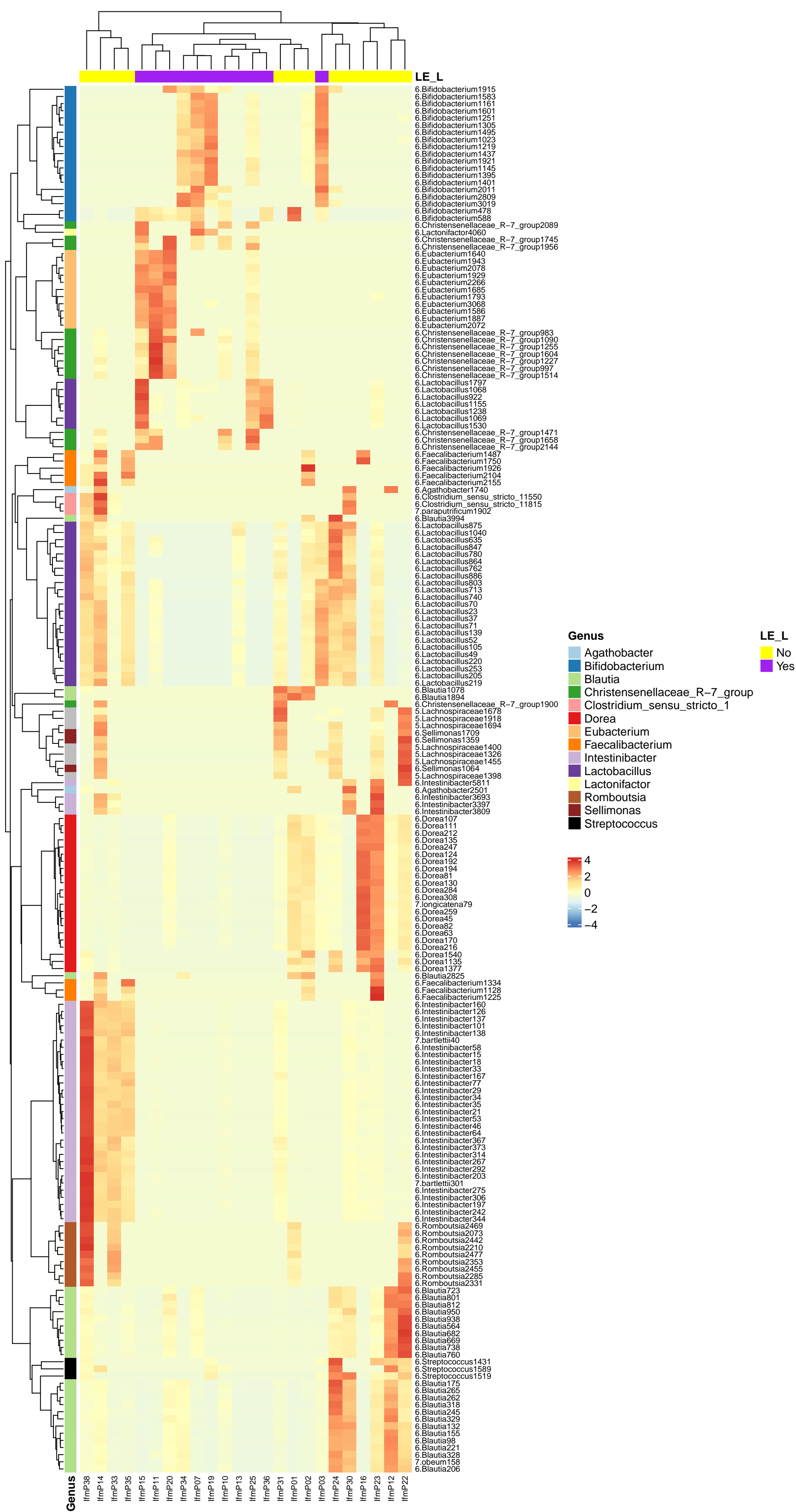

Supplement: Supplementary file 1 [file DataSheet_1.pdf]

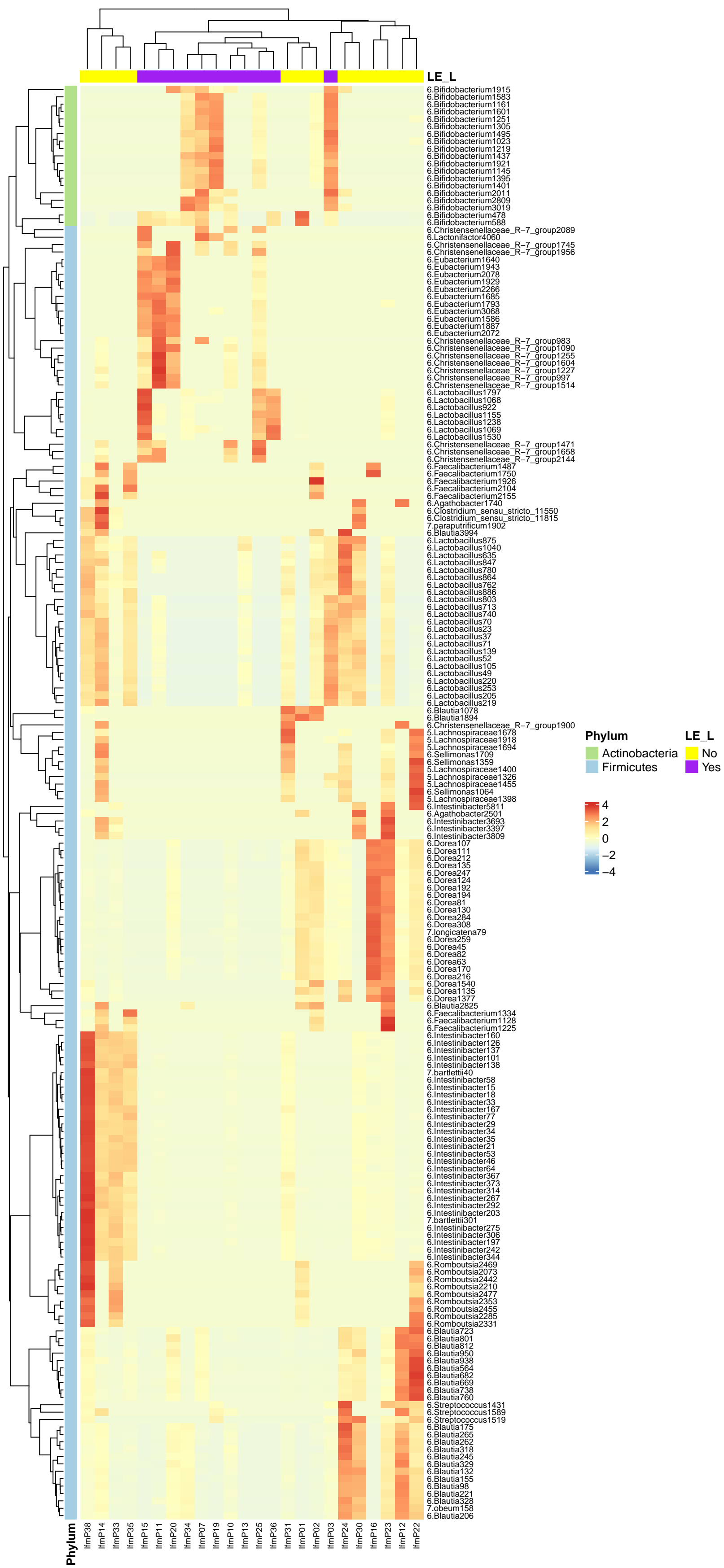

Supplement: Supplementary file 2 [file DataSheet_2.pdf]

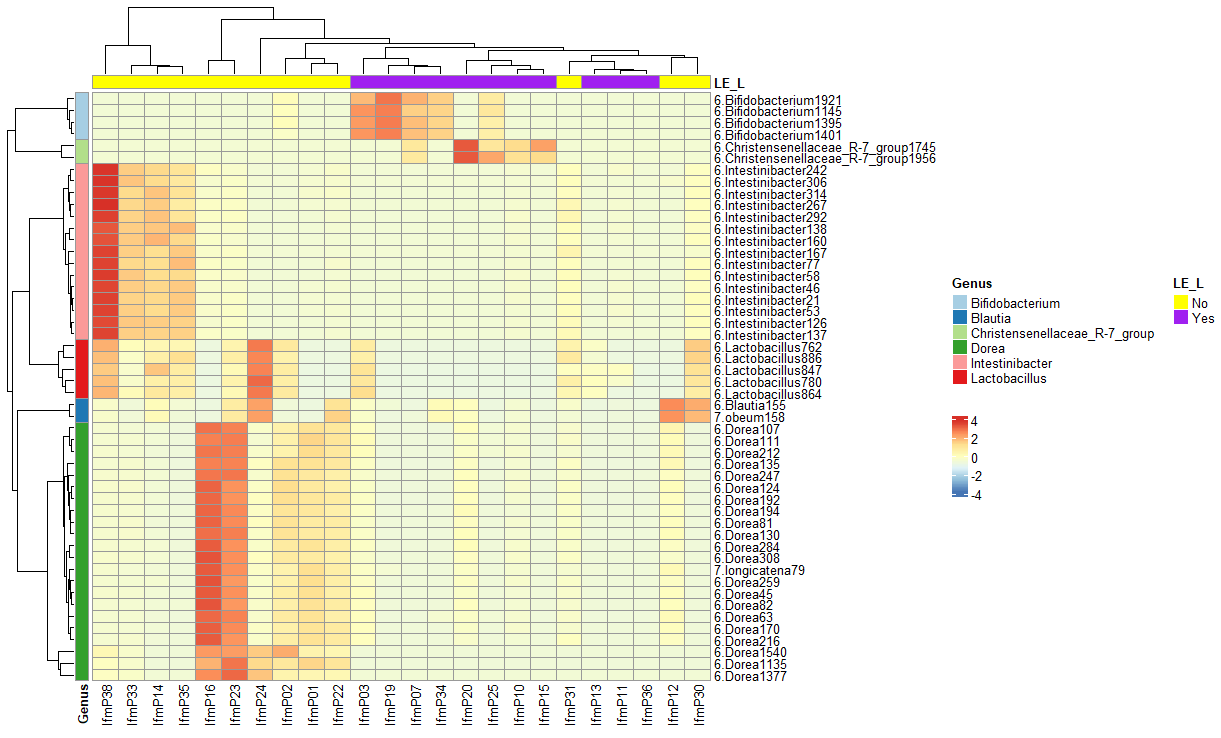

Supplement: Supplementary file 3 [file Image_1.png]

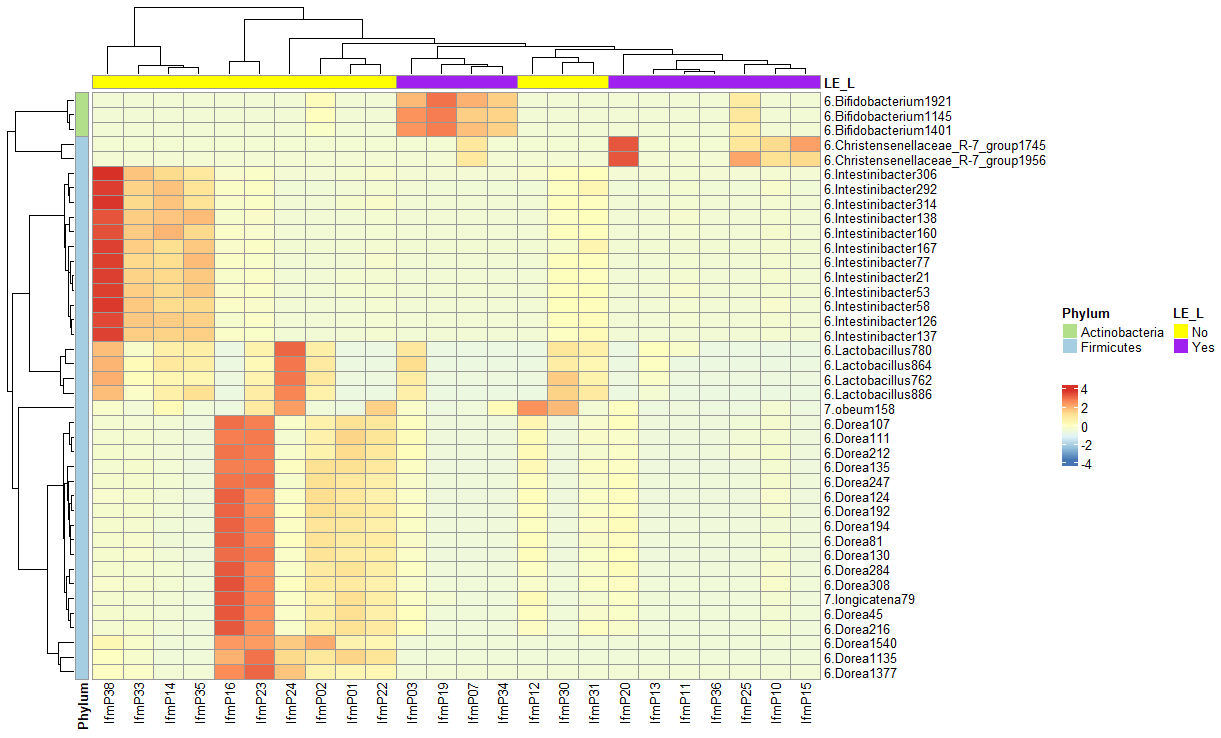

Supplement: Supplementary file 4 [file Image_2.png]

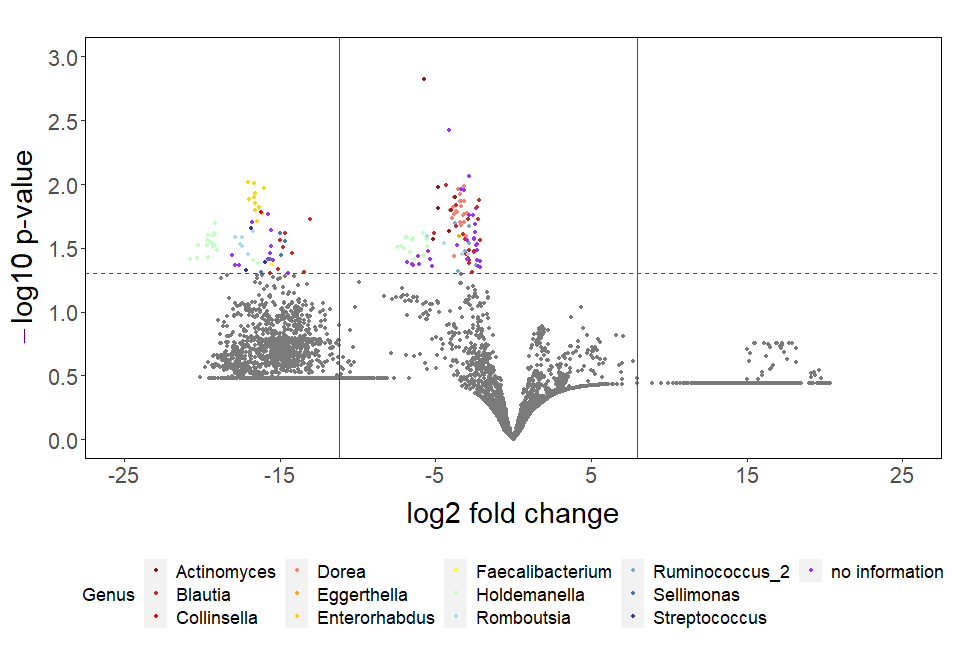

Supplement: Supplementary file 5 [file Image_3.png]

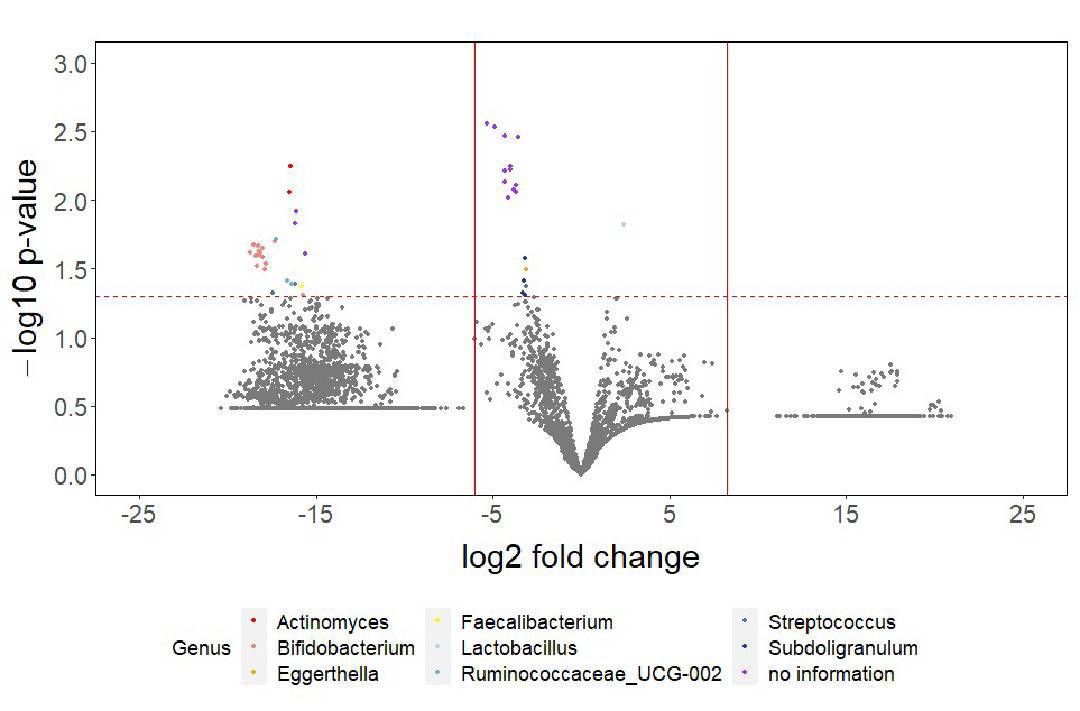

Supplement: Supplementary file 6 [file Image_4.jpeg]
